# Occurrence and Distribution of Pharmaceuticals and Their Transformation Products in Luxembourgish Surface Waters

**DOI:** 10.1021/acsenvironau.1c00008

**Published:** 2021-07-29

**Authors:** Randolph R. Singh, Adelene Lai, Jessy Krier, Todor Kondić, Philippe Diderich, Emma L. Schymanski

**Affiliations:** † Luxembourg Centre for Systems Biomedicine (LCSB), University of Luxembourg, 6 avenue du Swing, 4367 Belvaux, Luxembourg; ‡ IFREMER (Institut Français de Recherche pour l’Exploitation de la Mer), Laboratoire Biogéochimie des Contaminants Organiques, Rue de l’Ile d’Yeu, BP 21105, Nantes 44311 Cedex 3, France; § Institute for Inorganic and Analytical Chemistry, Friedrich-Schiller University, Lessing Strasse 8, 07743 Jena, Germany; ⊥ Administration de la gestion de l’eau, Ministère de l’Environnement, du Climat et du Développement durable, L-2918 Luxembourg, Luxembourg

**Keywords:** pharmaceuticals, surface water, suspect screening, HRMS, transformation products, cheminformatics, open source, nontarget screening

## Abstract

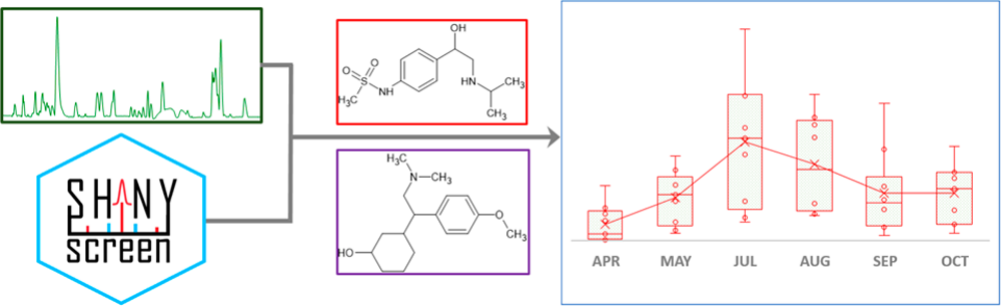

Pharmaceuticals and their transformation products (TPs) are continuously released into the aquatic environment via anthropogenic activity. To expand knowledge on the presence of pharmaceuticals and their known TPs in Luxembourgish rivers, 92 samples collected during routine monitoring events between 2019 and 2020 were investigated using nontarget analysis. Water samples were concentrated using solid-phase extraction and then analyzed using liquid chromatography coupled to a high-resolution mass spectrometer. Suspect screening was performed using several open source computational tools and resources including Shinyscreen (https://git-r3lab.uni.lu/eci/shinyscreen/), MetFrag (https://msbi.ipb-halle.de/MetFrag/), PubChemLite (https://zenodo.org/record/4432124), and MassBank (https://massbank.eu/MassBank/). A total of 94 pharmaceuticals, 88 confirmed at a level 1 confidence (86 of which could be quantified, two compounds too low to be quantified) and six identified at level 2a, were found to be present in Luxembourg rivers. Pharmaceutical TPs (12) were also found at a level 2a confidence. The pharmaceuticals were present at median concentrations up to 214 ng/L, with caffeine having a median concentration of 1424 ng/L. Antihypertensive drugs (15), psychoactive drugs (15), and antimicrobials (eight) were the most detected groups of pharmaceuticals. A spatiotemporal analysis of the data revealed areas with higher concentrations of the pharmaceuticals, as well as differences in pharmaceutical concentrations between 2019 and 2020. The results of this work will help guide activities for improving water management in the country and set baseline data for continuous monitoring and screening efforts, as well as for further open data and software developments.

## Introduction

The geography and history of Luxembourg have distinct implications on its environment and water quality: it borders Belgium, France, and Germany, and its rivers feed into the Rhine basin. Luxembourg has vineyards lining the Moselle River, agricultural activity in the north of the country, and a population largely centered in the capital, which together brings in a significant and varied chemical load into the environment. Previous studies have reported the presence of analgesics, antimicrobials, and estrogens in Luxembourgish surface water.^1−3^ Aside from providing data on the level of xenobiotics in Luxembourgish waters, these studies have also demonstrated that the presence of these chemicals is due to inputs from land use, accidental spillage, wastewater effluent, and long-range transport.^1,3−6^ Other studies have reported the measurement of 14 pesticides and their transformation products (TPs) in both surface water and drinking water.^3,7^ The Luxembourg Water Management Agency (Administration de la Gestion de l’Eau, hereafter AGE), in compliance with the European Union Water Framework Directive (WFD), monitors different organic contaminants in Luxembourgish surface water.^8^ Among the 92 compounds included in the targeted analysis performed by AGE, five are pharmaceuticals: carbamazepine, diclofenac, ibuprofen, ketoprofen, and lidocaine, while the rest the targeted organic contaminants are pesticides and related compounds.

As there are conceivably more pharmaceuticals than the five included in targeted monitoring that enter into the environment, it is important to determine which other pharmaceuticals may be present, to gain a more holistic idea of the pharmaceutical loading in Luxembourgish surface waters. The presence of pharmaceuticals in the aquatic environment poses a threat to human and environmental health due to exposure to either the pharmaceuticals themselves or their metabolites and TPs, which may still possess bioactivity.^9−11^ These chemicals have potential negative impacts on human health and the environment through different routes of exposures.^12,13^

There are many approaches to account for the presence of xenobiotics in the environment, but recently, increasing effort has been in the use of nontargeted analysis (NTA) and/or suspect screening using high-resolution mass spectrometry (HRMS) specifically to support risk assessment efforts and regulatory institutions.^14−16^ HRMS enables measurement of known pollutants, discovery of contaminants of emerging concern, as well as retrospective screening.^17^ However, setting up analyses, both experimentally and computationally, is no trivial matter. Despite these challenges, the information that can be obtained from such analyses has a wide breadth of utility, especially for environmental studies. NTA and suspect screening are effective techniques for the monitoring and discovery of xenobiotics in the aquatic environment.^17−20^ Nevertheless, the interpretation of HRMS data presents challenges that highlight the need for computational tools to enable the proper identification and annotation of the chemical components in environmental matrices.^21^

MetFrag (https://ipb-halle.github.io/MetFrag/)^22^ is an open source tool for compound identification, including *in silico* fragmentation, mass spectral matching, and metadata functions.^23,24^ MetFrag enables spectral matching with experimental data via the spectral library MassBank of North America (MoNA, https://mona.fiehnlab.ucdavis.edu)^25^ and prioritization using metadata from various sources. MetFrag first retrieves candidates by exact mass or molecular formula from one of many available compound databases. PubChem (https://pubchem.ncbi.nlm.nih.gov/)^26^ is an open chemistry database at the National Institutes of Health (NIH) containing more than 110 million compounds.^27^ While such a large database provides access to many chemicals, it can lead to (tens of) thousands of candidates per unknown when performing nontarget screening of hundreds of masses.^28^ For this work, an early version of PubChemLite was used, which contains ∼300,000 compounds selected to be highly relevant for environmental investigations based on annotation content, including information relevant for pharmaceuticals.^28,29^ PubChemLite has been shown to outperform other databases such as the whole of PubChem and CompTox for well-known chemicals^28^ and delivers important metadata that can be used during identification with MetFrag. PubChem and PubChemLite also contain information on environmental TPs contributed via the NORMAN Suspect List Exchange (https://www.norman-network.com/nds/SLE/).^28,30^ This information can be exploited programmatically during the environmental screening of hundreds of compounds, together with their transformation products.

Considering the previously reported presence of chemicals in Luxembourg’s environment^2,4−7^ and the widespread use of chemicals in daily life, a large number of compounds could be considered as potential environmental pollutants in Luxembourg. This work focuses on the presence of pharmaceuticals and known pharmaceutical TPs present in Luxembourg surface water systems using a mixture of instrumental measurements and cheminformatics approaches.

## Materials and Methods

### Sample Collection and Processing

**Figure 1:**
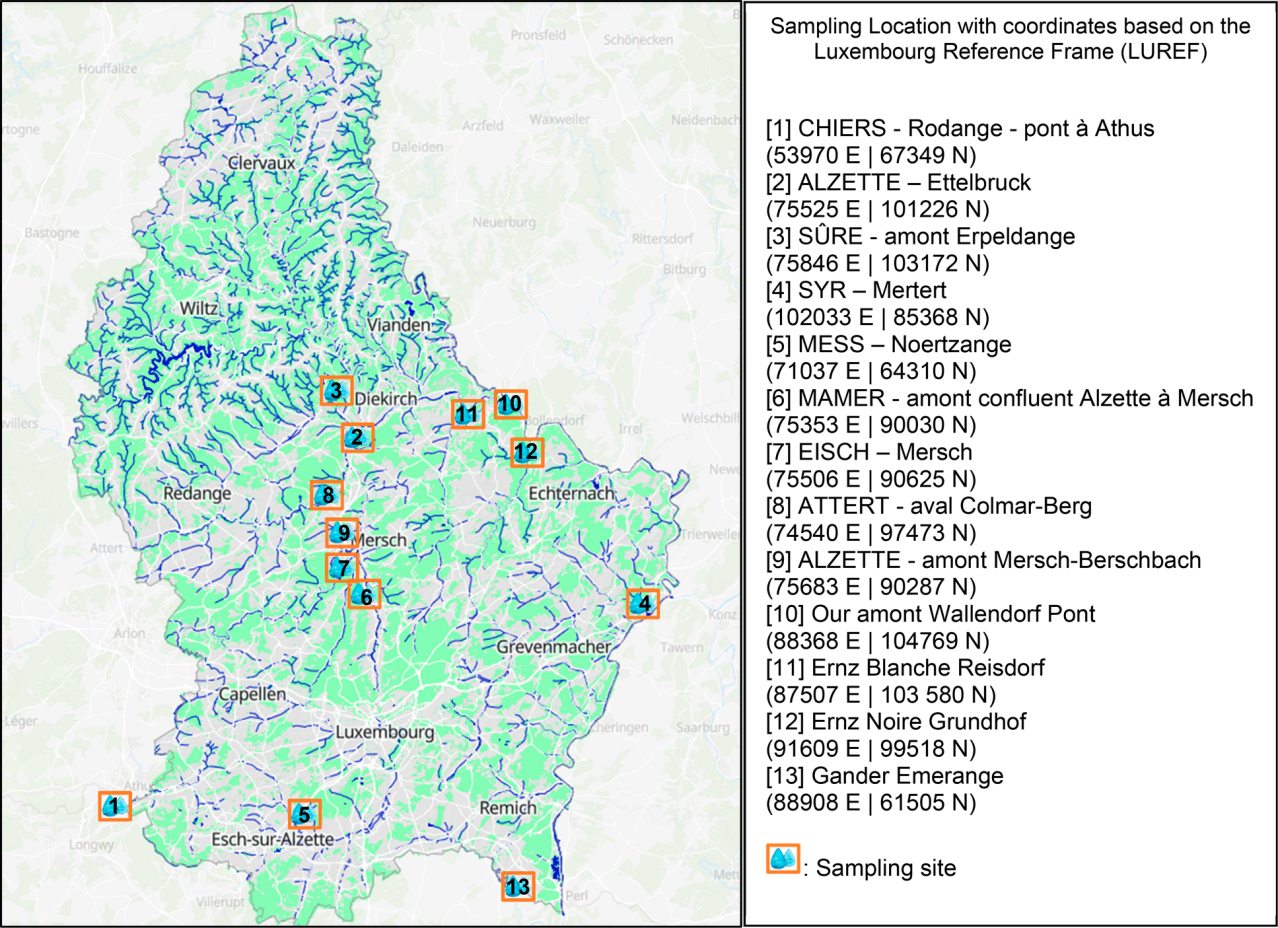
Sampling locations and their respective coordinates. Sampling locations 1–4 were sampled from 2019 to 2020; sampling locations 5–9 were sampled only in 2019, and sampling locations 10–13 were sampled only in 2020. Map generated using https://www.geoportail.lu/en/. Copyright MapTiler OpenStreetMap contributors.

Surface water samples (1 L) were collected every 4 weeks, whenever physically possible, from nine different locations in Luxembourg from April to November 2019 (Figure 1) and eight different locations from April to August in 2020 in accordance with the triannual sampling strategy employed at AGE. In this strategy, four locations monitored in compliance with the WFD are consistently sampled every 4 weeks (locations 1–4, Figure 1), while the other locations throughout Luxembourg are divided into three regions and are alternately sampled during a 3 year cycle. The samples were filled in 1000 mL amber glass bottles and stored for up to 1 week at 5 ± 3 °C in the dark until extraction. A method blank was prepared every month to account for potential contamination from sample handling using ultrapure water. Solid-phase extraction (SPE) was performed using Atlantic HLB SPE disks from Horizon (Salem, NH, USA) with a 47 mm diameter. The disks were conditioned twice for 1 min using acetonitrile and then twice for 1 min using Milli-Q water. The samples were pumped through each disk at a flow rate of roughly 30 mL/min, using the SPE-DEX 47900 system from Horizon (Salem, NH, USA). Sample loading was followed by washing the disks twice for 1 min with milli-Q water and drying by airflow for 15 min. The analytes were eluted for 1 min with cyclohexane, followed by an acetone elution for 1 min, then four times for 1 min with acetonitrile. After each elution step, the disks were air-dried for 1 min. The combined extracts were reduced to dryness under nitrogen flow in a water bath heated to 40 °C. The samples were resuspended in 2 mL of acetonitrile/water (10:90) by sonication for 5 min. Remaining particles were removed by passing the extracts through a 0.7 μm glass-fiber filter (Sartorius, Brussels, BE) into 2 mL amber glass LC-MS vials. The filtered extracts were stored at −20 °C until analysis.

### LC-HRMS Analysis

LC-HRMS analysis was performed on a Thermo QExactive HF mass spectrometer equipped with a Waters Acquity UPLC BEH C_18_ column (1.7 μm, 2.1 × 150 mm) using both positive and negative electrospray ionization with the following spray settings (positive/negative): sheath gas flow rate (45/60 arbitrary units, AU), auxiliary gas flow rate (10/25 AU), sweep gas flow rate (2/2 AU), spray voltage (3.5/3.6 kV), capillary temperature (320/300 °C), S lens RF (50/50 AU), and auxiliary gas temperature (300/370 °C). Mobile phases A (water with 0.1% formic acid) and B (methanol) were mixed using the following LC gradient starting at 90A/10B at 0 min, 90/10 at 2 min, 0/100 at 15 min, 0/100 at 20 min, 90/10 at 21 min, and ending with 90/10 at 30 min at a flow rate of 0.200 mL/min. The following data-dependent (dd-)MS2 settings (in display order of instrumental acquisition method) were used: resolution (120,000 at *m*/*z* 200), automatic gain control (AGC) target (1.0 × 10^6^), maximum injection time (IT): (70 ms), and scan range (*m/*z = 60–900). For the selected ion monitoring of dd-MS2/ddSIM, the following were used: resolution (30,000 at *m*/*z* 200), AGC target (5.0 × 10^5^), maximum IT (70 ms), loop count (5), Top N (5), isolation window (1.0 Da), (N)CE (30). Lastly, the following dd settings were used: minimum AGC target (8.0 × 10^3^), intensity threshold (1.1 × 10^5^), apex trigger (4–6 s), exclude isotopes (On), and dynamic exclusion (10.0 s). The instrument was calibrated and optimized every time an analysis was performed using manufacturer settings to ensure consistent performance throughout the 2 year study. A 100 μg/L standard mixture containing cyclizine, desipramine, nylidirin, amiloride, dibucaine, dothiepin, ethambutol, etofyline, mefruside, phenazone, phentermine, sulfamoxole, sulfamethoxazole, and metoclopramide obtained from Dr. Herbert Oberacher was used to monitor instrument performance between analyses.^31^

### Suspect Screening

Suspect screening was performed using two suspect lists. The first list contains 816 unique pharmaceutical compounds (Supporting Information, Table S1 CNS “Caisse Nationale de Santé” Suspects, also available on the NORMAN Suspect List Exchange, NORMAN-SLE)^30,32^ that were curated from the Luxembourgish National Health Fund’s “List of marketed medications in Luxembourg”.^33^ These drugs have marketing authorization in Luxembourg from the Ministry of Health and are therefore potentially in use domestically. For suspect screening, MS-ready SMILES of these compounds were obtained via the EPA CompTox Chemistry Dashboard’s batch search function.^34,35^ Using MS-ready SMILES as a structural identifier ensures that the structure being used for data analysis is consistent with what is measured by the mass spectrometer and at the same time remains traceable within online chemical databases.^35^

The second suspect list consists of 82 pharmaceutical TPs. These TPs were derived from two sources: PubChem^28^ and a recent study by Anliker et al.^18^ From PubChem, TPs were obtained from the transformations table of a given compound (where available) using R scripts^36^ written to programmatically download transformation product information.^37^ The TP information in PubChem originates from the NORMAN Suspect List Exchange.^28,30^ Sixty-seven TPs were extracted from PubChem in this way (coming from a total of 53 parents—44 parents were on the original CNS list of 816 parent compounds, while the remaining nine parents are actually themselves TPs with reciprocal transformations). The remaining 15 TPs were obtained from Anliker et al.^18^ Curation of the final suspect list involved deduplication and multiple steps of interconversion between chemical identifiers (e.g., CAS to PubChem CID, InChIKey to CID) using PubChem’s Identifier Exchange Service^38^ to facilitate compound comparisons and ensure that the final list of 82 TPs was unique. Then, the final SMILES (“parent SMILES” in PubChem terms, “MS-ready” SMILES in CompTox terms) were retrieved. More information and the full R code are available in the Supporting Information and on GitLab as a Jupyter Notebook.^39^

Prescreening was performed using Shinyscreen (https://git-r3lab.uni.lu/eci/shinyscreen),^40^ an open source and freely available mass spectral processing software developed in house to extract MS1 data and the associated MS2 events and spectra. Detailed information on its functions, installation, and usage can be found by following the link provided above. The following settings for extraction and automatic quality control were used: coarse precursor *m*/*z* error (±0.5 Da), fine precursor *m*/*z* error (±2.5 ppm), extracted ion chromatogram (EIC) *m*/*z* error (±0.001 Da), retention time (*t*_r_) tolerance (±0.5 min), MS1 intensity threshold (1.0 × 10^5^), MS2 intensity threshold relative to MS1 peak intensity (0.05), signal-to-noise ratio (3), and retention time shift tolerance (±0.5 min). Note that for suspect screening where *t*_r_ information is not available, the *t*_r_ tolerance on the MS1 level is still provided as a setting to Shinyscreen, but the whole chromatogram is screened. For suspect or target chemicals where the *t*_r_ is known from previous analysis (and provided in the input files), this threshold is then applied (e.g., in the suspect confirmation efforts). The “retention time shift” setting at the MS2 level controls the tolerance with regards to alignment of the MS1 and MS2 signals. Features that passed QC through manual curation including peak shape, peak width, peak intensity, and alignment of the MS1 and MS2 peaks were then analyzed using MetFrag to achieve tentative identifications. Scripts used for this work are available on GitLab.^39^ PubChemLite was used as database, available as a local .csv file,^29^ to find chemicals that match the exact mass (within 5 ppm) of the suspect pharmaceutical. Both in silico fragmentation (mzabs = 0.001, frag_ppm = 5) and experimental MS/MS matching through MoNA records (built within MetFrag) were performed to obtain the fragmenter (scoring term 1) and MoNA (scoring term 2) scores. Metadata were also collected for the candidates by querying the database for patent count (scoring term 3), number of PubMed references (scoring term 4), PubChem annotation count (scoring term 5), pharmacology and biochemistry information (scoring term 6), and drug and medication information (scoring term 7). The latter two scoring terms assist in the interpretation of the results where multiple relevant candidates occur per mass, as described recently elsewhere,^28^ as well as in the retrieval of classification information (mentioned below). Candidates were ranked and given a score per category normalized to 1 and then added together to obtain the max_score, with the highest possible score = 7. A more detailed explanation of the parameters used is available elsewhere.^28,41^ Annotation confidence levels were determined using the scheme described by Schymanski et al.^42^ Level 2a compounds were assigned when the MoNA score was greater than or equal to 0.9. Level 1 identifications were achieved using authentic standards and the ENTACT mixtures,^43^ available in-house and analyzed using the same chromatographic method used for sample analysis. The ENTACT mixtures were obtained from participation in the EPA’s non-targeted analysis collaborative trial.^43^ Retention times were considered a match if the difference was less than 0.2 min. The compound classification for the compounds identified was obtained by consulting PubChem’s “Drug and Medication Information” section, based on a specific drug’s therapeutic use or function. Level 3 confidence was given for compounds with max_score > 6.0 but with MoNA scores less than 0.9 (103 compounds); however, the scope of the paper has been limited to level 2a and level 1 chemicals at this stage due to their higher confidence.

**Figure 2:**
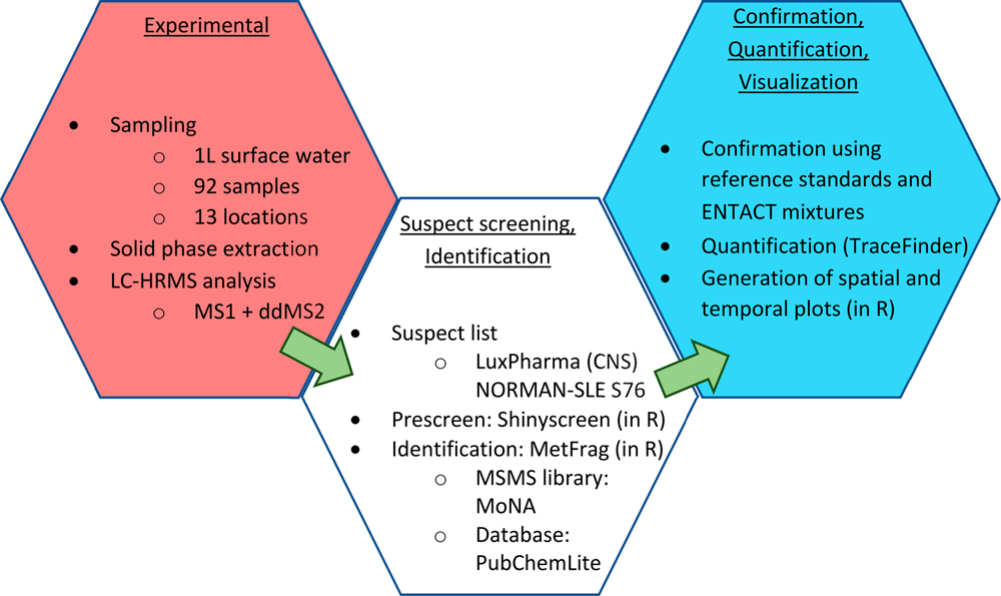
Flow diagram of the experimental and data processing workflow employed in this work.

Where reference standards were available, the concentration of the pharmaceuticals was quantified using an external calibration curve ranging from 1 to 1000 μg/L spanning the linear dynamic range for the compounds quantified. Tracefinder (Thermo Scientific, version 5.1) was used for automatic peak integration and generation of the calibration curve. Concentrations below 1 μg/L were reported to be below the quantifiable range. With the exception of nonanedioic acid, where the blank comprised <1% of the signal and was subtracted, no interference from the blank was observed for the other analytes identified in this work. After compound identification and quantification, a spatiotemporal analysis was performed to determine whether there were specific areas with higher pharmaceutical loading and/or monthly variability. The concentration of pharmaceuticals in surface waters is influenced by many factors such as matrix, precipitation, volume, wastewater effluent discharge, as well as significant changes in cross-border mobility in 2020 due to the pandemic (a dominating factor in Luxembourg where half of the workforce live outside the country). As a result, the spatial and temporal comparisons are limited to uncorrected concentration values here and should be interpreted accordingly. For spatial analysis, the median concentration of the identified compound across the different months was calculated and presented by sampling year. For temporal analysis, the median concentration of the identified compound across locations 1–4 was used, as these locations were sampled consistently irrespective of sampling year. A boxplot was also constructed to see which pollutants are consistently high and to show the difference in detected concentrations between 2019 and 2020. Heat maps and boxplots were generated using custom-made, openly accessible scripts in R.^44^ Results were compared to pharmaceuticals found in the Meuse (Belgian and Dutch section) and Rhine (German section) rivers, which all have Luxembourgish rivers as tributaries. A simplified version of the workflow employed in this work is presented in Figure 2.

## Results and Discussion

### Identification of Pharmaceuticals and Their TPs

After LC-HRMS analysis coupled with cheminformatics tools was performed, 88 compounds were confirmed at level 1 confidence; 86 of these could be quantified. Amantadine and 8-hydroxyquinoline concentrations were too low to be quantified. A further six compounds were identified at level 2a. These results are summarized in Tables 1 and 2. Among the detected compounds, only seven were detected in both positive and negative ionization: diclofenac, fluconazole, irbesartan, losartan, niflumic acid, oxazepam, and valsartan (further identifiers are provided in the Supporting Information, Tables S1 and S2). In terms of pharmaceutical class, many of the compounds identified in this work belong to drugs for the management of heart-related diseases (15), psychoactive drugs (15), antimicrobials (eight), and drugs for the management of pain (eight). All five chemicals monitored by AGE were also detected in this study. The number of analytes, including both levels 1 and 2a, found per location in this study ranged from 23 compounds (July 2020) to 52 compounds (May 2019). Thirty-eight pharmaceuticals were detected at least 90% of the time, accounting for 40% of the total compounds identified in this study.

**Table 1:**
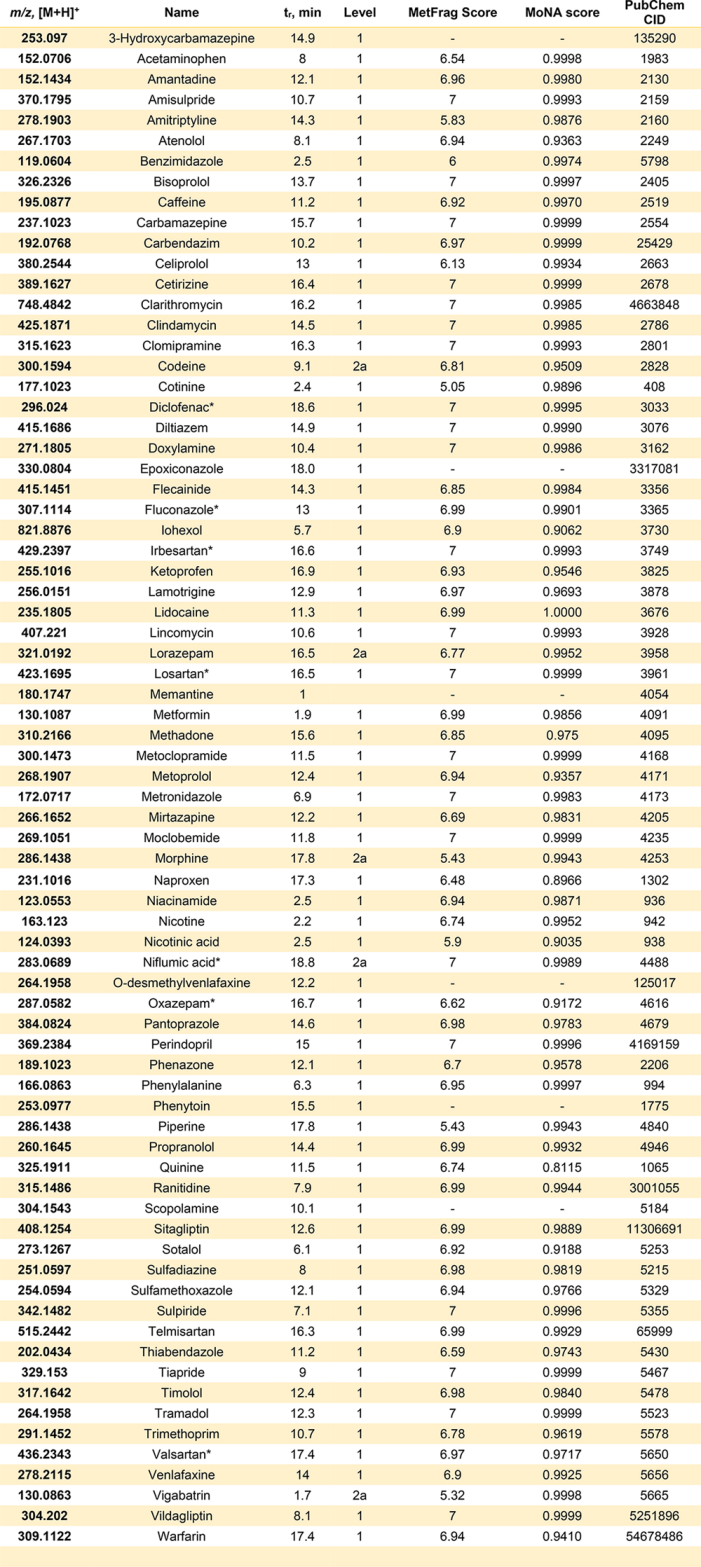
Summary of Pharmaceuticals and Pharmaceutical Transformation Products in Positive Mode Found in Luxembourgish River Water^a^

a An extended version with structural information is available in the Supporting Information Table S2, Pharma IDs. *t*_r_ = retention time. *Found in both positive and negative modes.

**Table 2:**
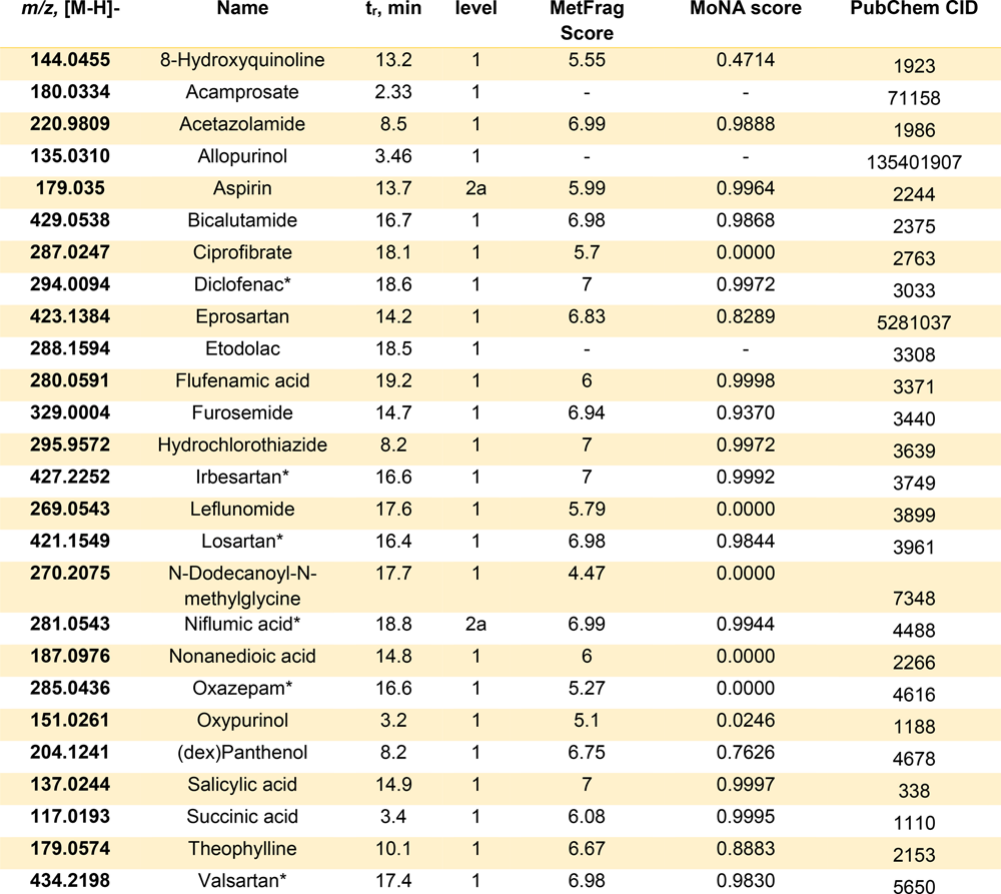
Summary of Pharmaceuticals and Pharmaceutical Transformation Products in Negative Mode Found in Luxembourgish River Water^a^

a An extended version with structural information is available in the Supporting Information Table S2, Pharma IDs. *t*_r_ = retention time. *Found in both positive and negative modes.

Two TPs (3-hydroxycarbamazepine and *O*-desmethylvenlafaxine) were identified with level 1 confidence, whereas 12 TPs were identified at level 2a confidence and are listed including their parent compounds in parentheses: 4-acetamidoantipyrine (metamizole), 4-aminoantipyrine (metamizole), clopidogrel carboxylic acid (clopidrogel), cotinine (nicotine), D617 (verapamil), ritalinic acid (methylphenydate), fenofibric acid (fenofibrate), flucytosine (emtricitabine), guanylurea (metformin), morphine (codeine), N4-acetylsulfamethoxazole (sulfamethoxazole), 4-hydroxydiclofenac (diclofenac). Flucytosine on its own is used as an antifungal agent, whereas morphine can be used as the parent compound for pain management. In addition, two TPs (2-hydroxycarbamazepine and 10,11-dihydroxycarbamazepine) were tentatively identified (level 3) during the parent pharmaceutical screening because they were isobaric with some parent pharmaceuticals.

### Spatiotemporal Distribution of Pharmaceuticals in Luxembourg

**Figure 3:**
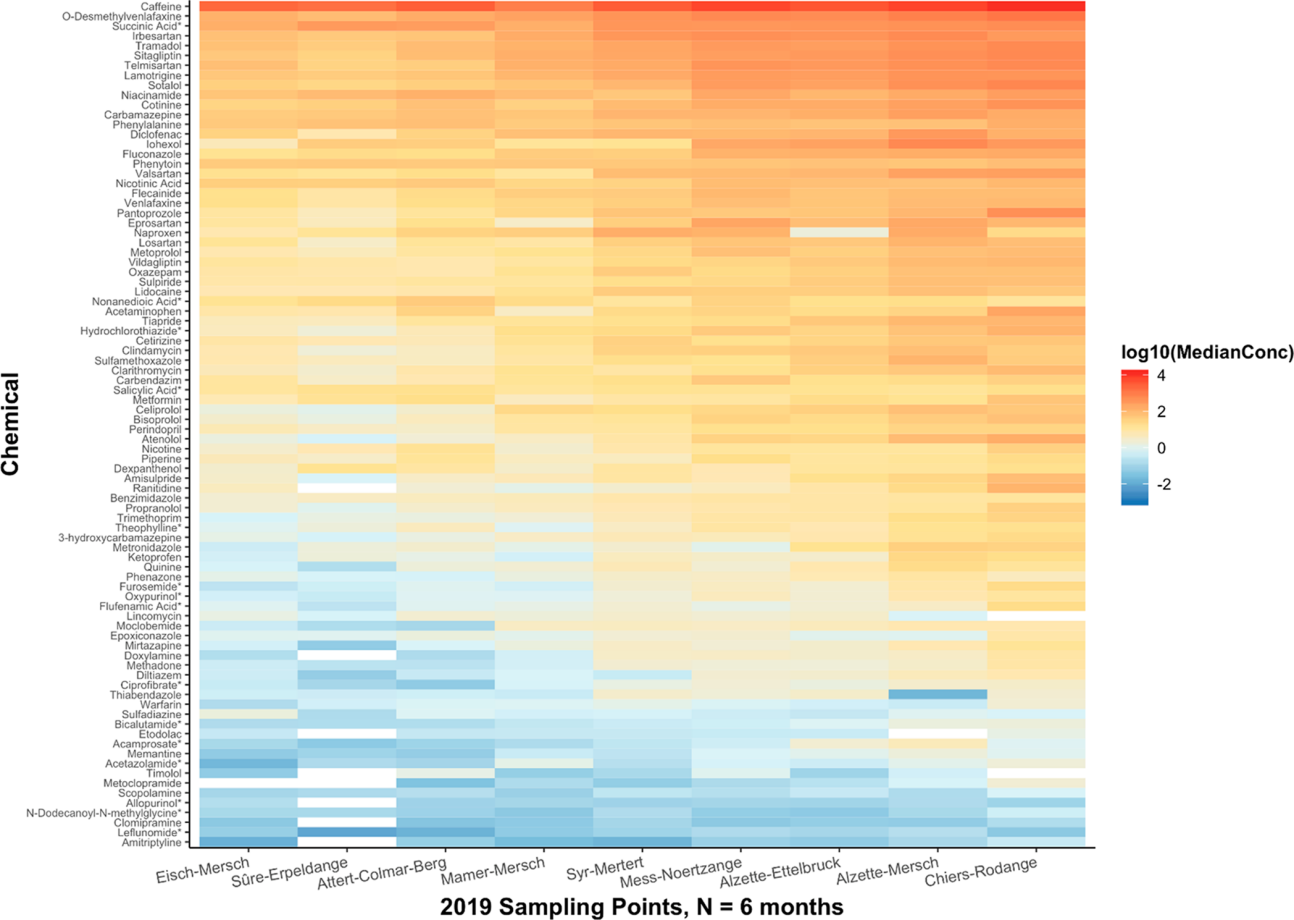
Spatial heat map showing median concentration values (original units: ng/L) per compound measured per sampling location over 6 months in 2019, plotted using a base-10 logarithmic scale. Median values were calculated across the concentrations measured over the relevant months of sampling for the respective compound and location. Zero-value median concentrations are indicated by gray-shaded boxes. White boxes indicate that there were no concentration values within the quantification range. All compounds were measured in positive mode except for those marked with an asterisk, which were measured in negative mode.

**Figure 4:**
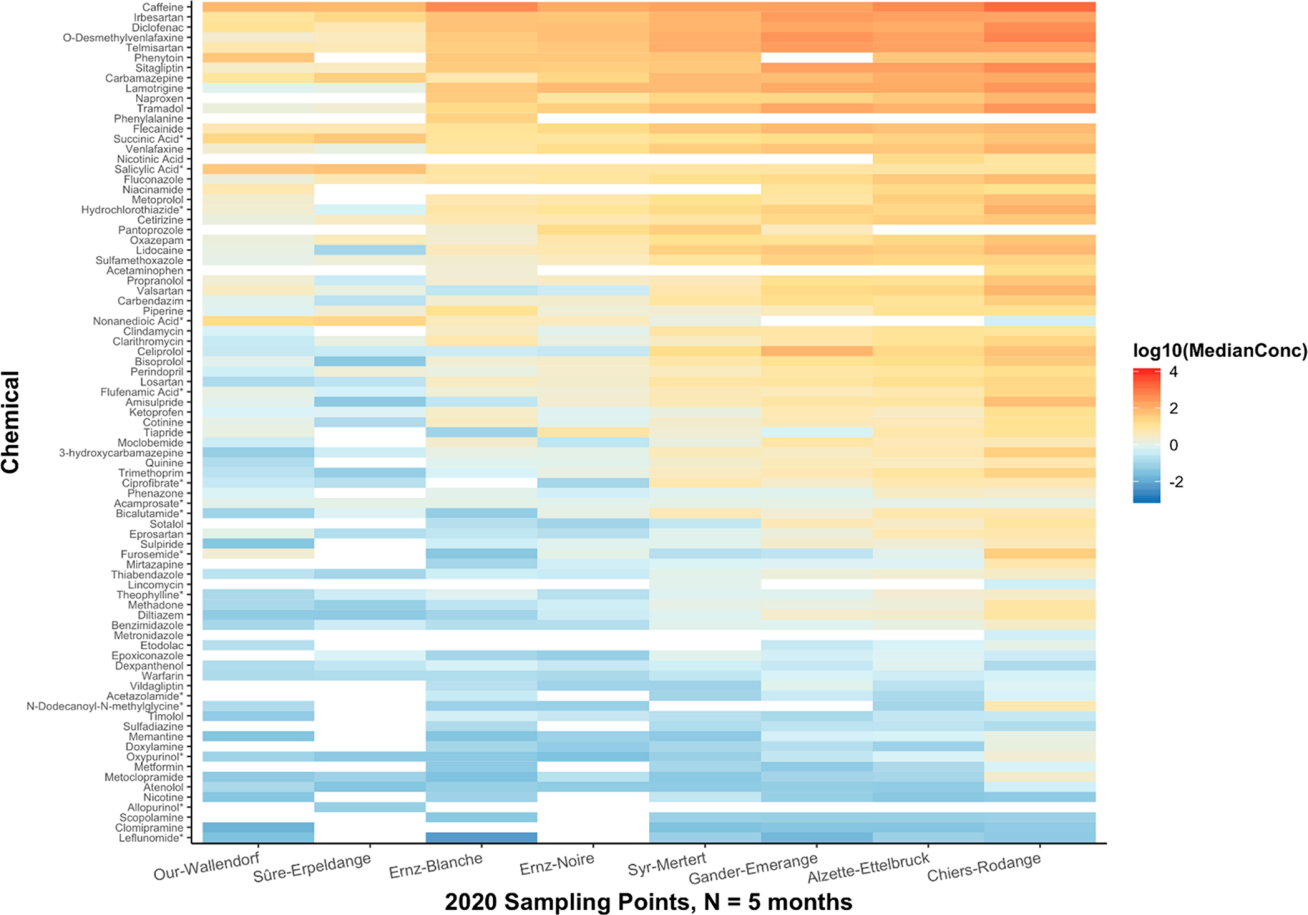
Spatial heat map showing median concentration values (original units: ng/L) per compound measured per sampling location over 5 months in 2020, plotted using a base-10 logarithmic scale. Median values were calculated across the concentrations measured over the relevant months of sampling for the respective compound and location. Zero-value median concentrations are indicated by gray-shaded boxes. White boxes indicate that there were no concentration values within the quantification range. All compounds were measured in positive mode except for those marked with an asterisk, which were measured in negative mode.

**Figure 5:**
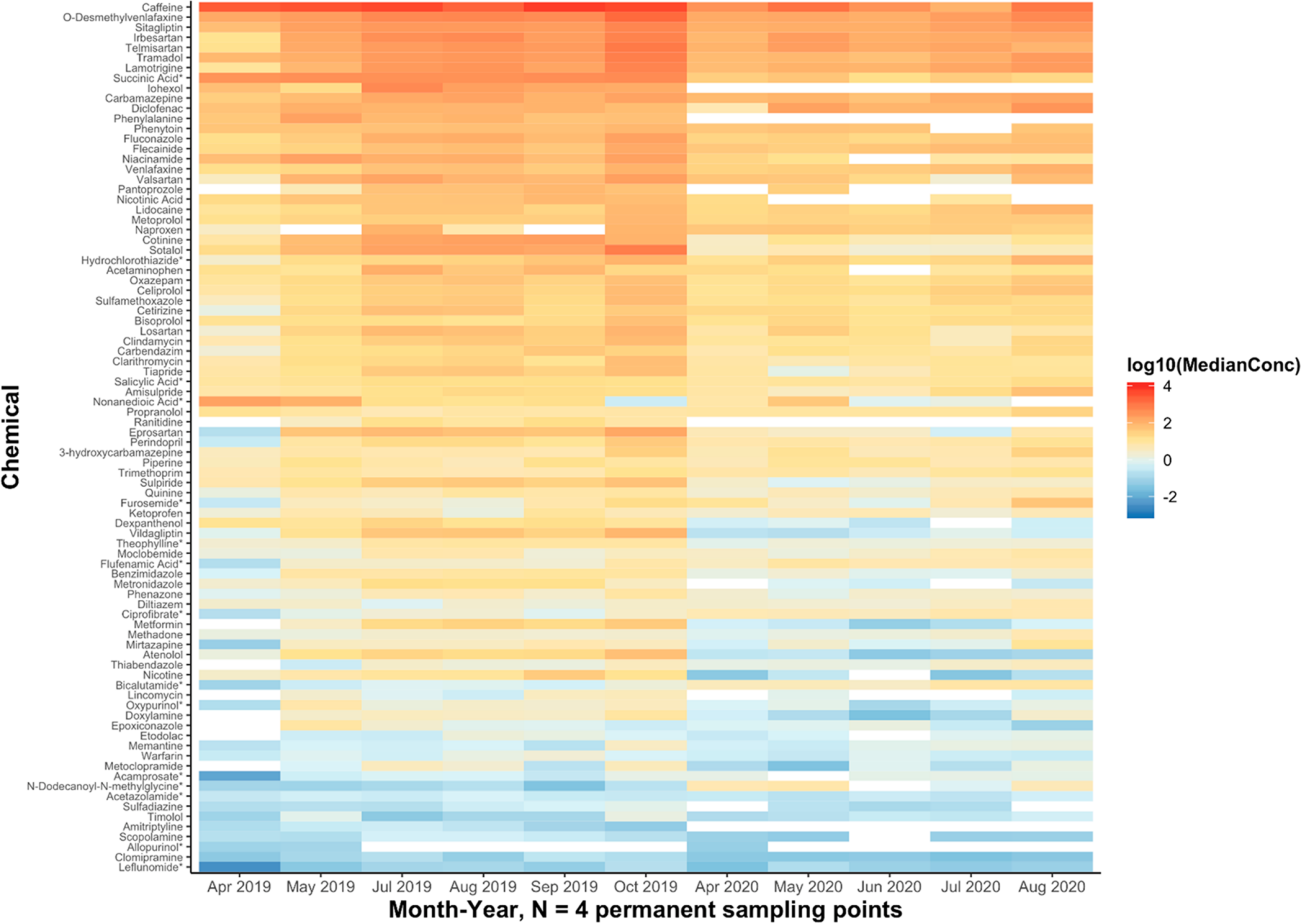
Temporal heat map showing median concentration values (original units: ng/L) per compound measured per sampling month–year plotted using a base-10 logarithmic scale. Median values were calculated across the concentrations measured at the four permanent sampling locations for the respective compound and month–year. Zero-value median concentrations are indicated by gray-shaded boxes. White boxes indicate concentration values that were below the respective quantification range, which were therefore discarded from median calculation. All compounds were measured in positive mode except for those marked with an asterisk, which were measured in negative mode.

The median concentrations of the different compounds identified in this work, irrespective of ionization polarity, were plotted to generate the spatial (*N* = 6 time points for 2019, *N* = 5 time points for 2020) and temporal (*N* = 4 sampling points) heat maps presented in Figures 3, 4, and 5, respectively. Note that only locations 1–4 were sampled consistently between 2019 and 2020, in compliance with the WFD requirements; thus only data from these locations were used for the temporal analysis. Locations 5–9 were only sampled during 2019, whereas locations 10–13 were sampled in 2020. Tables S3 (negative mode) and S4 (positive mode) in the Supporting Information summarize the individual concentration of each pharmaceutical quantified from 2019 to 2020 from each location. The spatial heat maps (Figures 3 and 4) for both 2019 and 2020 consistently show that Chiers-Rodange-pont à Athus (location 1, Figure 1), followed by Alzette-Ettelbruck (location 2, Figure 1) and Alzette-Mersch-Berschbach (location 9, Figure 1) that have higher levels of pharmaceutical contamination.

Among the pharmaceuticals found were antihypertensive drugs. In 2019, sotalol and telmisartan were the antihypertensive drugs detected at the highest concentration. In contrast, irbesartan was detected to have the highest concentration during 2020, followed by telmisartan. All three drugs were found to be highest in location 1 (Chiers-Rodange-pont à Athus) followed by location 2 (Alzette-Ettelbruck), irrespective of sampling year. Clarithromycin and clindamycin, on the other hand, were the antimicrobials detected with the highest concentration in 2019, respectively. However, in 2020, sulfamethoxazole and trimethoprim were the highest detected antimicrobials. These drugs are known to be used together for the treatment of bacterial infections. Locations 1 and 2 consistently showed the highest concentrations of the above-mentioned antimicrobials irrespective of year.

The Chiers river receives effluent from the Petange wastewater treatment plant (capacity: 70,000 population equivalents), which is close to the Chiers-Rodange-pont à Athus sampling point. This proximity is likely one of the reasons why Chiers-Rodange-pont à Athus was found to have the highest concentration of pharmaceuticals within this study. In comparison, both Alzette-Ettelbruck and Alzette-Mersch-Berschbach are downstream of the Beggen wastewater treatment plant^45^ (capacity: 210,000 population equivalents), which receives sewage from Luxembourg City, the biggest and most populated city in Luxembourg. Despite the bigger capacity, both sampling points are not as close to the source as the Chiers location and thus may experience dilution. The lowest median concentrations for the pharmaceuticals quantified in this study were found at Eisch-Mersch (2019, location 7 in Figure 1), Sûre-amont Erpeldange (2020, location 3, Figure 1), and Our amont Wallendorf Pont (2020, location 10, Figure 1). Pharmaceutical compounds found in this study such as acetaminophen, caffeine, carbamazepine, clarithromycin, salicylic acid, and valsartan have been described before as markers of sewage or wastewater discharge into surface water,^46,47^ further supporting the impact of wastewater effluents in Luxembourgish rivers.

**Figure 6:**
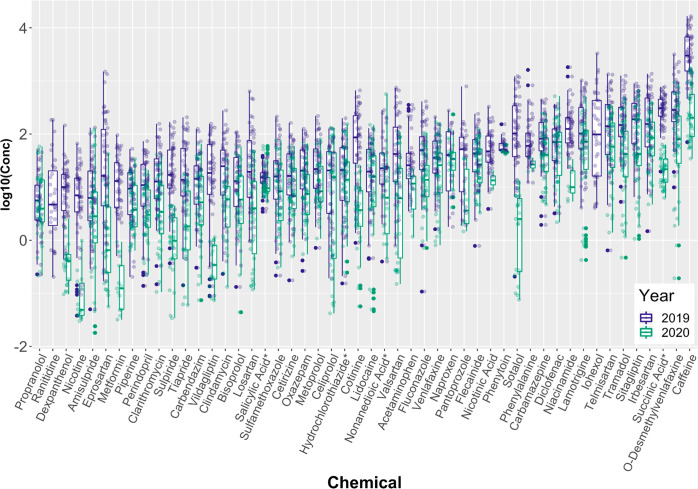
Boxplots showing the range of concentrations (original units: ng/L) measured for the top 50 highest concentration pharmaceutical chemicals across all months and sampling locations in 2019 and 2020, plotted using a base-10 logarithmic scale. Concentration values that were below the respective quantification ranges were excluded. All chemicals were measured in positive mode.

Figures 3–6 show the dynamic nature of pharmaceutical contamination in surface water, demonstrating that aquatic organisms in these rivers are exposed to varying mixtures over time. Since recent studies have highlighted the ecological risks associated with exposure to mixtures in surface water systems,^48,49^ this work helps show how suspect screening may support the identification of more chemicals in surface waters and thus help improve the ecological risk assessment of mixtures in future works.

The stimulant caffeine, antidepressant metabolite *O*-desmethylvenlafaxine, antihypertensive drugs irbesartan and telmisartan, the antidiabetic drug sitagliptin, and the opioid analgesic tramadol were among the most concentrated pharmaceuticals found in Luxembourgish surface waters (Figures 3 and 4) in both 2019 and 2020. From a temporal point of view (Figure 5), the highest median concentrations of the pharmaceuticals were detected in September and October of 2019 and are consistently lower during the spring. The most visually obvious differences between the two sampling years include (1) amytriptyline, iohexol, phenylalanine, and ranitidine only detected at quantifiable levels in 2019 and (2) decreases in the median concentrations of dexpanthenol, metformin, nicotine, sotalol, and vildagliptin. As an example, metformin had median concentrations of 3.0 ng/L (May) to 39 ng/L (October) in 2019, much higher than the highest detected median concentration of metformin in 2020 (0.62 ng/L in August 2020). Dexpanthenol is a drug used for prophylactic purposes; both metformin and vildagliptin are drugs used for managing diabetes, sotalol is for the management of arrhythmia, while nicotine relates to smoking. A juxtaposition of data from 2019 and 2020 is presented as boxplots in Figure 6, showing the general decrease in many pharmaceutical concentrations in 2020 (green boxes). For simplicity, only the top 50 pharmaceuticals ranked by median concentration are presented. Some of the most notable drops in detected concentration were observed for dexpanthenol, nicotine, metformin, and sotalol. The individual concentrations of the analytes per sampling location and time are summarized in Tables S3 and S4 in the Supporting Information.

### Factors That Affected Pharmaceutical Concentrations in Luxembourg

Interestingly, lower median concentrations of the pharmaceuticals were measured in 2020 compared to those measured in 2019 (as shown in Figure 6), which may be partially due to the reduced presence of cross-border workers during the pandemic. COVID-19 has brought on a major shift in working practices, as more people were advised and allowed to work remotely. In Luxembourg, a major part of the workforce comprises cross-border workers (approximately 206,000 people in 2019).^50^ This translates to an approximately 25% decrease in the daytime population, which may translate to reduced pharmaceutical loading in the sewage system. Two interesting features in Figure 6, also apparent in Figure 5, are the detections of iohexol and ranitidine in 2019 but not in 2020. Iohexol is a radiocontrast agent used for medical imaging. Due to the COVID-19 pandemic, there was a significant decrease in medical procedures for noncommunicable diseases, including radio imaging.^51^ This decrease may explain why iohexol was not detected at a quantifiable level in 2020 despite having the sixth highest median concentration in 2019. Ranitidine use in the EU, on the other hand, was discontinued in 2020 because of the suspected carcinogen *N*-nitrosodimethylamine, an impurity present in ranitidine drugs.^52^ It is interesting to see how changes in drug usage are abruptly reflected in their detection in the environment.

Changes in precipitation had been reported to affect contaminant levels in water, generally increasing with increased precipitation due to factors such as runoff and combined sewer overflow.^53^ Compared to the long-term average (1981 to 2010), both 2019 and 2020 experienced a decrease in the annual precipitation (Table 3). For the samplings months that were studied in both 2019 and 2020 (April, May, July, and August), 2020 showed the lowest amount of precipitation, which may have contributed to the lower concentration of pharmaceuticals detected. While there was not sufficient data available in this study to fully account for all factors influencing the concentration such as population, precipitation, matrix effects, and extraction recoveries, these results reveal interesting trends that will be the subject of further work.

**Table 3:**
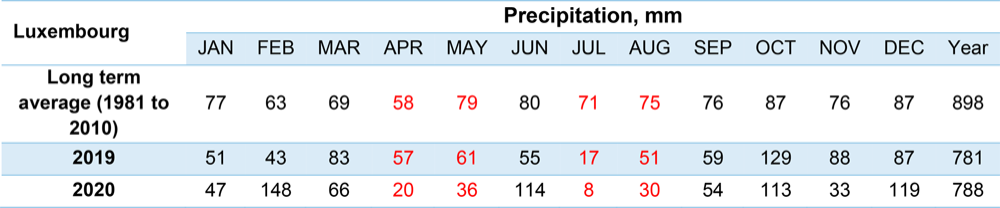
Precipitation Data for Luxembourg^a^

a Source: https://www.meteolux.lu.

While the Chiers flows into the Meuse River and the Alzette flows into the Sauer River (eventually leading into the Rhine), both rivers contribute to the chemical load that eventually ends up in the North Sea. Several studies have determined the presence of pharmaceuticals in the Meuse and Rhine rivers. A 2010 study by ter Laak et al. reported compounds such as caffeine, carbamazepine, lidocaine, and iohexol as some of the more concentrated pharmaceuticals in their study of the Rhine, with sulfamethoxazole as the most abundant antimicrobial.^54^ The same study also found antihypertensive drugs such as atenolol, metoprolol, and sotalol. Despite being apart by almost a decade, similar trends can be observed in Luxembourgish waters. Later studies of different parts of the Rhine and Meuse rivers reported similar pharmaceuticals;^55,56^ however, in some studies, the antidiabetic drug metformin and its TP guanylurea were found to be the most abundant pharmaceutical in surface water samples.^55,57,58^ While metformin was also quantified in this study, the median concentration only ranks 44^th^ over both years among the pharmaceuticals found. Higher levels of the antidiabetic drug sitagliptin, fifth most abundant, were detected in Luxembourg. The two drugs differ in their mode of regulating sugar in the body.

### Challenges in Compound Identification

The presence of isobars, isomers, and in-source fragments complicates the identification of chemicals in HRMS data, sometimes even leading to these analytes to be excluded from HRMS analysis.^59,60^ Several cases of isobars were encountered in this work including (a) acetaminophen and 1,2,3,6-tetrahydrophthalimide, (b) salicylic acid, 3-hydroxybenzoic acid, and 4-hydroxybenzoic acid, (c) piperine, morphine, and etodolac, (d) cocaine and scopolamine, (e) tramadol and *O*-desmethylvenlafaxine, and (f) phenytoin, 2-hydroxycarbamazepine, and 3-hydroxycarbamazepine. While cases a–d were easily resolved using authentic standards, cases e and f introduced specific challenges. Tramadol (parent compound) and *O*-desmethylvenlafaxine (TP of venlafaxine) are constitutional isomers whose extracted ion chromatogram shows two unresolved peaks that are both annotated by MetFrag as tramadol (due to tramadol’s higher metadata scores). Using standards, the first peak (12.2 min) was ultimately assigned to be *O*-desmethylvenlafaxine, while the second peak (12.4 min) was tramadol. In order to quantify both compounds, the peaks had to be manually integrated to avoid integrating the two peaks as one compound.

**Figure 7:**
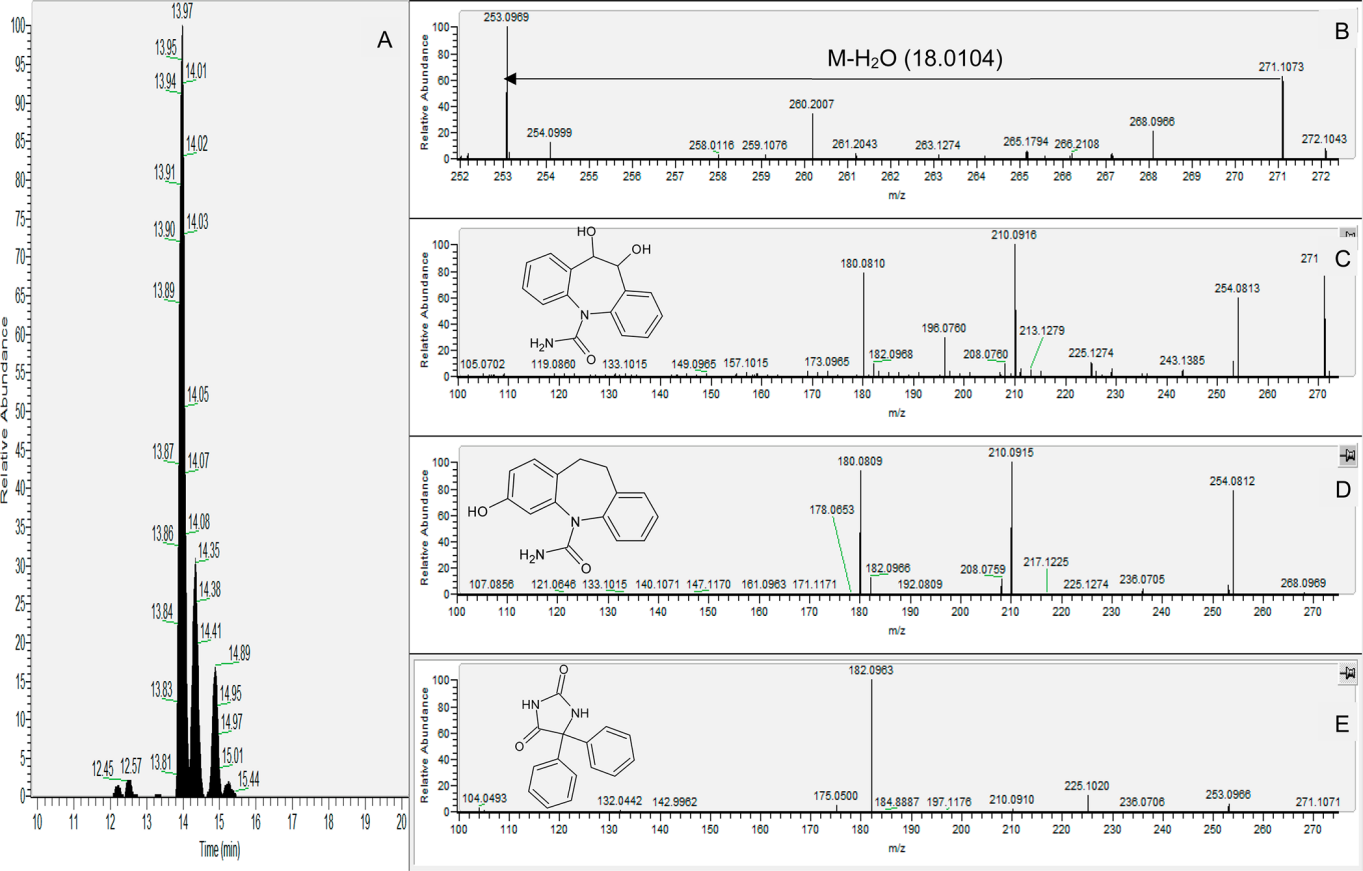
(A) Extracted ion chromatogram of *m*/*z* = 253.0969 in a surface water sample showing three distinct peaks. (B) MS1 spectrum of the 13.97 peak showing a higher peak that may have lost water to produce the 253.0969 peak. (C) MS2 spectrum of *m*/*z* = 271.1073 (potentially 10,11-dihydroxycarbamazepine, structure on the same pane) showing similar fragments to the MS2 fragments of 3-hydroxycarbamazepine standard (structure on the same pane); see (D). (E) MS2 spectrum of the phenytoin standard (structure on the same pane).

For the suspect screening of phenytoin, three prominent peaks (*t*_r_: 13.95, 14.31, and 14.85 min) were observed in the positive mode extracted ion chromatogram of *m*/*z* 253.0972 within 5 ppm error (Figure 7A). Looking at the structure of phenytoin, the absence of chiral carbons renders the possibility of diastereomers, which could explain the presence of multiple peaks, invalid. Analysis of the phenytoin standard showed that this compound elutes at 15.53 min, thus not matching any of the three peaks being investigated. Further inspection using MetFrag and database matching suggested that the second and third peaks belong to the positional isomers 2-hydroxycarbamazepine and 3-hydroxycarbamazepine, metabolites of the anticonvulsant carbamazepine. The *t*_r_ matching using a standard confirmed that the peak at 14.85 min is indeed 3-hydroxycarbamazepine, while the peak at 14.31 min can be assigned as 2-hydroxycarbamzepine (level 3), despite the lack of standards, due to the similarity of its mass spectrum with 3-hydroxycarbamazepine. However, the first and biggest peak proved to be challenging. Inspection of the MS1 spectrum at 13.95 min shows another peak with *m*/*z* 271.1075 (mass difference equivalent to the loss of water, Figure 7B) can be found whose MS2 spectrum is very similar to the 253.0972 peaks at 14.31 and 14.85 min (Figure 7C,D). Using these pieces of information, it can be suggested that the 253.0972 peak is potentially an in-source fragment of 271.1075. Using 271.1075 as the precursor ion, MetFrag suggests that the peak is potentially 10,11-dihydroxycarbamazepine (MoNA score: 0.8340) or phenytoin acid (MoNA score: 0.8076), which are TPs of carbamazepine and phenytoin, respectively. The presence of the 210.0915 and 180.0811 fragments, which match fragments of other carbamazepine metabolites, and the earlier elution suggesting that the molecule is more polar than the monohydroxylated analogs, supports the tentative identification of the 13.95 min peak as 10,11-dihydroxycarbamazepine (level 3).

One case that needs further inspection is the stereoisomers vidarabine and adenosine, which are impossible to separate using the chromatographic method employed in this study. While there are reports on the utility of ion mobility to discriminate between stereoisomers, it is still to be tested whether such resolution is practically achievable.^61−63^ Published collisional cross sections of vidarabine (156.4 Å^2^ for [M + H]^+^) and adenosine (156.9 Å^2^ for [M + H]^+^) measured on the same instrument are available, revealing a difference of only 0.5 Å^2^ or 0.3%, which is too close to distinguish currently within the typical resolving power of ion mobility spectrometers.^64,65^

This study documents suspect screening efforts thus far for pharmaceuticals and their known TPs as a starting point for further understanding pharmaceutical levels in Luxembourgish surface waters. Other activities looking into different chemical classes such as pesticides,^66^ industrial chemicals, and other emerging pollutants are ongoing. The continuous analysis of surface water using HRMS as part of the routine monitoring efforts will enable retrospective screening^67,68^ for newly identified contaminants that may impact local surface water quality and biota, such as the effect observed by city runoff on coho salmon.^69^ Very recently, a portable HRMS setup for surface water monitoring was demonstrated to enable real-time pollutant analysis,^70^ which would be interesting to consider in future efforts pending availability. This study reports primarily level 1 and 2a identifications due to the hard filter of MoNA score of >0.9 applied during the MetFrag analysis. Other tentative identifications have been communicated with AGE, and these, along with more detailed trend analysis as more temporal data points are collected, can be investigated in future works as resources allow. Quantification efforts could be further improved using the list of pharmaceuticals identified in this work as a target list, as well as investing in isotopically labeled standards (which was beyond the scope of the current works, as target analysis is performed by AGE). Finally, as experimental databases increase in size and coverage, the ability to screen for more compounds with higher confidence with these open source methods such as the one presented here will also increase, highlighting the need for the community at large to continue to contribute to publicly available databases.

One main factor limiting TP suspect screening is the lack of available information in open databases that is standardized and thus suitable to be extracted consistently and reproducibly to form meaningful suspect lists. Of the 816 parent compounds on the CNS list, only 44 had associated TP information (i.e., one or more TPs) that could be extracted from PubChem as performed in this study. Certainly, there are far more pharmaceutical metabolites/TPs than those that are identified here, but this information is not yet available in a readily extractable form suitable for an automated workflow within PubChem (the efforts within the NORMAN Suspect List Exchange have just commenced recently).^28,66^ As more information is added and as more environmental transformation studies are performed and deposited in a FAIR (findable, accessible, interoperable and reusable) manner,^71^ the ability to screen for TPs in an automated fashion would also increase and support further research efforts.

## Supporting Information Available

The Supporting Information is available free of charge at https://pubs.acs.org/doi/10.1021/acsenvironau.1c00008. The suspect list used in this work is available online as LUXPHARMA (S76) on Zenodo (DOI: 10.5281/zenodo.4587356), CompTox (https://comptox.epa.gov/dashboard/chemical_lists/LUXPHARMA, PubChem (https://pubchem.ncbi.nlm.nih.gov/classification/#hid=101), and NORMAN-SLE (https://www.norman-network.com/nds/SLE/). The data (as .mzML files) are available as data set MSV000087190 from the GNPS MassIVE repository (https://massive.ucsd.edu/ProteoSAFe/static/massive.jsp), citable under DOI: 10.25345/C5D81C and accessible via ftp://massive.ucsd.edu/MSV000087190/ and https://massive.ucsd.edu/ProteoSAFe/dataset.jsp?accession=MSV000087190. Both Shinyscreen (https://git-r3lab.uni.lu/eci/shinyscreen/) and MetFrag (http://ipb-halle.github.io/MetFrag/) are open source; additional support scripts mentioned are available from the ECI GitLab repository (https://git-r3lab.uni.lu/eci/pubchem). All code used to run MetFrag in the command line using R, generate the Transformation Products suspect list, and plot Figures 3–6 is available via https://git-r3lab.uni.lu/adelene.lai/additional_si_luxpharma_singh_et_al. All other code and databases used as part of MetFrag identification are likewise openly available (links inline throughout this article).

Tables of CNS suspects, pharma IDs, negative mode, positive mode, positive concentration, and the original file names and their corresponding names in this paper (the original file names were kept to allow traceability to the original sample files stored locally at the University of Luxembourg) (XLSX)
